# Using quantitative single molecule localization microscopy to optimize multivalent HER2-targeting ligands

**DOI:** 10.3389/fmed.2023.1064242

**Published:** 2023-04-17

**Authors:** Devin L. Wakefield, Ottavia Golfetto, Raphael Jorand, Sunetra Biswas, Kassondra Meyer, Kendra N. Avery, Cindy Zer, Eliedonna E. Cacao, Steven J. Tobin, Ian J. Talisman, John C. Williams, Tijana Jovanovic-Talisman

**Affiliations:** Department of Cancer Biology and Molecular Medicine, Beckman Research Institute, Duarte, CA, United States

**Keywords:** HER2, meditope, valency, single molecule localization microscopy, trastuzumab, pertuzumab

## Abstract

**Introduction:**

The progression-free survival of patients with HER2-positive metastatic breast cancer is significantly extended by a combination of two monoclonal antibodies, trastuzumab and pertuzumab, which target independent epitopes of the extracellular domain of HER2. The improved efficacy of the combination over individual antibody therapies targeting HER2 is still being investigated, and several molecular mechanisms may be in play: the combination downregulates HER2, improves antibody-dependent cell mediated cytotoxicity, and/or affects the organization of surface-expressed antigens, which may attenuate downstream signaling.

**Methods:**

By combining protein engineering and quantitative single molecule localization microscopy (qSMLM), here we both assessed and optimized clustering of HER2 in cultured breast cancer cells.

**Results:**

We detected marked changes to the cellular membrane organization of HER2 when cells were treated with therapeutic antibodies. When we compared untreated samples to four treatment scenarios, we observed the following HER2 membrane features: (1) the monovalent Fab domain of trastuzumab did not significantly affect HER2 clustering; (2) individual therapy with either trastuzumab or (3) pertuzumab produced significantly higher levels of HER2 clustering; (4) a combination of trastuzumab plus pertuzumab produced the highest level of HER2 clustering. To further enhance this last effect, we created multivalent ligands using meditope technology. Treatment with a tetravalent meditope ligand combined with meditope-enabled trastuzumab resulted in pronounced HER2 clustering. Moreover, compared to pertuzumab plus trastuzumab, at early time points this meditope-based combination was more effective at inhibiting epidermal growth factor (EGF) dependent activation of several downstream protein kinases.

**Discussion:**

Collectively, mAbs and multivalent ligands can efficiently alter the organization and activation of the HER2 receptors. We expect this approach could be used in the future to develop new therapeutics.

## 1. Introduction

Roughly 20% of breast cancers exhibit overexpression and/or gene amplification of human epidermal growth factor receptor 2 (HER2). These cases are typically associated with aggressive disease and poor outcomes (1–6). The patient outcomes have been significantly improved by therapeutic monoclonal antibodies (mAbs) targeting HER2 (7–13). The first mAb therapy against HER2 to be approved by the U.S. Food and Drug Administration (FDA) was trastuzumab; it binds close to the transmembrane domain of HER2, on an extracellular region (domain IV), and it may inhibit HER2 homodimerization (14). At least four mechanisms of action have been suggested for trastuzumab: it induces internalization and degradation of HER2, activates antibody-dependent cellular cytotoxicity (ADCC), prevents shedding of the HER2 extracellular domain, and/or inhibits downstream signaling (15–17). While trastuzumab may benefit patients through one or more of these effects, a significant challenge in the clinic has been both intrinsic and acquired resistance (15, 18). Understanding the manifestation of these mechanisms is critical to guide alternative treatment strategies.

The combination of mAbs have been suggested as an approach to improve clinical outcomes in several cancers (13, 19, 20). In fact, patients with HER2-positive breast cancer benefited when they received a combination of trastuzumab and pertuzumab plus chemotherapy (10–13, 21). Pertuzumab binds to the extracellular domain II (the dimerization domain) of HER2 and inhibits its heterodimerization with other HER family members (22, 23). Pertuzumab may prevent HER2/HER3 heterodimerization, activate ADCC, and inhibit downstream signaling (9, 17, 22, 23). When pertuzumab was combined with trastuzumab, the density of HER2 on the plasma membrane was reduced (24, 25). The enhanced efficacy of trastuzumab plus pertuzumab may also be associated with two additional effects: an increase in ADCC (17) and inhibition of both homo- and hetero-dimerization of HER2, which subsequently attenuates the HER2 signaling cascade (26–28). Local differences in HER2 membrane patterning in different cell types (29–33) also point toward functional differences, which are likely relevant to the therapeutic response. Details that would shed light on these mechanisms remain poorly understood at the molecular level, and accordingly, our group and others have been studying how therapeutic agents affect HER2 molecular organization as a means to gain additional insight to ultimately improve clinical outcomes.

To this end, we have developed methods for quantitative single molecule localization microscopy (qSMLM). This approach is designed to count detected receptors, report on the spatial patterns of these receptors, and define their regional heterogeneity. In our prior study, we observed significant changes in HER2 spatial organization when cultured cells were treated with the chemotherapeutic agent paclitaxel and targeted inhibitor afatinib (31). We have also extended HER2 single molecule localization microscopy (SMLM) imaging to patient specimens (32). Importantly, in cultured cell lines and pre-treatment patient biopsies, we observed that HER2 density and clustering appear to correlate with therapy sensitivity/response (33). Herein, we have applied qSMLM to study in more detail how HER2 patterning is affected by different antibody-based agents, either alone or in combination. In addition to studying clinical therapies, we probed a set of pre-clinical reagents known as meditopes. These small, peptide-based molecules bind to a unique site within the Fab arm of the clinically relevant antibody cetuximab (that targets epidermal growth factor receptor, EGFR). This site has also been engineered into other mAbs which we have named meditope-enabled mAbs (memAbs) (34). MemAbs retain their affinity, selectivity, and function (35–38). Importantly, this technology allows us to add unique functionality to mAbs, including “non-covalent crosslinking” through multivalent meditopes. Here, we observe that the valency of meditope-based reagents modulates the spatial patterns and activity of HER2 in the presence of a trastuzumab memAb. These results indicate that reagent valency may be exploited to develop novel antibody-based therapeutics.

## 2. Materials and methods

### 2.1. Coverslip preparation

Twenty-five-millimeter #1.5 coverslips (Warner Instruments) were cleaned and coated with fibronectin-like engineered protein [25 μg/ml in phosphate buffer saline (PBS), pH 7.4, Sigma] as described before (32).

### 2.2. Cell culture

MDA-MB-468 and SK-BR-3 cells (originally obtained from the American Type Culture Collection, ATCC) were cultured in Phenol Red-free Dulbecco’s Modified Eagle Medium (DMEM) supplemented with 10% fetal bovine serum, 1 mM sodium pyruvate, 100 units/ml penicillin, 100 units/ml streptomycin, and 2 mM L-alanyl-L-glutamine. MDA-MB-453 cells (also from ATCC) were cultured in DMEM-F12 media supplemented with 10% fetal bovine serum, 0.5 mM sodium pyruvate 100 units/ml penicillin and 100 units/ml streptomycin. BT-474 cells were cultured as described previously (32).

### 2.3. HER2-paGFP plasmid construct

A perbB2-EGFP pcDNA3.1(+) plasmid was purchased from Adgene (plasmid # 39321). This plasmid was used for iterative site directed mutagenesis to mutate the EGFP coding region into paGFP. The following primer pairs were used for iterative site directed mutagenesis to alter five amino acids in the EGFP coding region of the plasmid:

Primer pair 1

5’ CCCACCCTCGTGACCACCTTTAGTTACGGCGTGCAG TGCTTC 3’

5’ GAAGCACTGCACGCCGTAACTAAAGGTGGTCACGAG GGTGGG 3’

Primer pair 2

5’ GAACGGCATCAAGGCGAACTTCAAGATCC 3’

5’ GGATCTTGAAGTTCGCCTTGATGCCGTTC 3’

Primer pair 3

5’ GACAACCACTACCTGAGCCATCAGTCCAAACTGAG CAAAG 3’

5’ CTTTGCTCAGTTTGGACTGATGGCTCAGGTAGTGG TTGTC 3’

After each round of site directed mutagenesis, the DNA was transformed into BP5alpha competent cells (Biopioneer) and plated on LB/ampicillin agar plates at 37°C. Single colonies were selected from the plate and amplified in 10 ml LB culture to purify plasmid DNA to be used for the next round of site directed mutagenesis. After the final round of SDM was complete, the plasmid was sent for Sanger sequencing at the City of Hope Integrative Genomics Core.

### 2.4. Antibodies, antibody fragments, and fluorescent dye conjugation

Pertuzumab and trastuzumab (Genetech) were clinical grade. Trastuzumab memAb I83E (referred to here as trastuzumab memAb, or TmemAb) and wild type trastuzumab (for ADCC experiments) were prepared similarly as described before (34).

The Fc used for control studies was obtained from a papain digest of clinical trastuzumab. The Fc was purified by application of the papain digest material to first a protein L column (GE, 5 ml) with collection of the flow through. The flow through material was concentrated and further purified by gel filtration chromatography (GE, superdex 10/300 GL). The fractions were concentrated and stored at −80°C.

Fab trastuzumab was generated through the digestion of the clinical trastuzumab with immobilized papain (Pierce) and purified by reverse purification with protein A (GE Healthcare) and SEC on a HiLoad 16/600 pg Superdex 75 column (GE Healthcare).

MemAbs were labeled with Alexa Fluor 647 (AF647) dyes presenting an N-hydroxysuccinidimidyl ester (NHS) group for protein conjugation. A solution with 4–6 x excess of dye dissolved in dimethyl sulfoxide was mixed with a solution of 1 mg/ml of TmemAb in PBS, pH 7.4, and 0.02 M NaHCO_3_. The conjugation reaction solution was placed on a rotator for 30 min at room temperature and quenched with 1.5 M hydroxylamine (pH 8.5) for 10 min. Unconjugated dye was removed by passing the solution through a size exclusion chromatography column (Bio-Rad, Hercules, CA, USA) while any potential aggregates were removed by passing labeled Ab through a 300-kDa concentrator. Measurements from a NanoDrop 1000 (Thermo) were used to calculate (with respect to the dye correction factor) the final concentration and degree of labeling for each fluorescent antibody. Approximately one dye per antibody (degree of labeling ∼1) was obtained in all cases for antibodies labeled with AF647. These conditions are desired as an increased degree of labeling has been reported to decrease affinity for trastuzumab (39).

### 2.5. M2Fc and M4Fc

M2Fc contains the meditope sequence fused to the N-terminus of the Fc using a flexible 37 amino acid sequence (Supplementary methods). For the M4Fc moiety, the meditope sequence is placed at both N and C-termini using flexible 39 and 30 amino acid linkers respectively (Supplementary methods). Each were produced in insect cells. Specifically, baculovirus encoding each were produced in TNI insect cells according to the manufacturer’s protocol by transfecting M2Fc or M4Fc expression vectors and propagating the virus three times (Expression Systems). TNI cells were seeded in ESF921 media at a density of 1e6 cells/ml and allowed to grow overnight. The following day, high titer baculovirus containing the M2Fc or M4Fc expression vectors was added to the TNI cells at a MOI of 50 and the cells were allowed to produce protein. After three days, cells were separated from the media by centrifugation and media containing M2Fc or M4Fc was placed over a protein A column for purification. M2Fc or M4Fc in PBS was further purified using fast protein liquid chromatography.

### 2.6. Antibody dependent cell-mediated cytotoxicity (ADCC) assay

Antibody dependent cell-mediated cytotoxicity assays were conducted using a luciferase reporter-based core kit according to manufacturer instructions (G7010, Promega). SK-BR-3 cells were used as the target cell line and seeded at 5,000 cells per well in a 96-well white, tissue-culture treated plate in DMEM with 10% FBS one day prior to conducting the ADCC assay. The next day, media was replaced with 4% low IgG serum in RPMI media, according to kit protocol. Dilutions of listed antibodies were made at 1:2 starting at 1 nM antibody in the presence or absence of 10 nM M4Fc and added to cells along with the effector cells provided. Antibodies at 1 nM, with or without M4Fc, were also added to wells containing no target or effector cells as a negative control. Cells were incubated at 37^°^C for 6 h prior to adding luciferase substrate. Luminescence was measured on a Synergy 4 multi-mode microplate reader (BioTek) with a 0.5 s integration time. Each experiment was repeated at least three times with three technical replicates per experiment.

### 2.7. Transfection and antibody treatment of cells for imaging

MDA-MB-468 cells were transiently transfected 48 h after plating on coverslips using Jet Prime (PolyPlus) according to the manufacturer’s instructions. For steady-state measurements, cells were washed with PBS at 37°C and fixed with 4% (w/v) paraformaldehyde and 0.2% (w/v) glutaraldehyde (EMS, Cat# 157-8 and 16019, respectively, Hatfield, MA, USA) in PBS for 30 min at room temperature; fixation was quenched with 25 mM glycine for 10 min as described before (32).

For Ab/Ab fragment treatments, 24 h after HER2-paGFP transfection, MDA-MB-468 cells were washed with media and treated with: 20 nM Fab trastuzumab, 10 nM trastuzumab, 10 nM pertuzumab, or 10 nM trastuzumab combined with 10 nM pertuzumab in media for 10 min. Cells were washed again with warm media and fixed/quenched as described above. For multivalent meditope treatment, MDA-MB-468 cells transfected with HER2-paGFP were first incubated with meditope-enabled trastuzumab memAb for 10 min. After a quick warm media wash, cells were incubated with 10 nM Fc, 10 nM M2Fc, 20 nM M2Fc, or 10 nM M4Fc in media for indicated times and fixed. To assess the effect of multivalent meditopes on endogenous HER2, BT-474 cells were incubated with 10 nM TmemAb-AF647, alone or premixed with 10 nM multivalent meditope (i.e., M2Fc and its variants or M4Fc), for 10 min at 37°C and subsequently fixed as described above. Alternatively, cells were stained with 10 nM TmemAb-AF647 postfixation as indicated. All incubations were performed at 37°C in the cell culture incubator.

About 0.1 μm TetraSpeck beads (Life Technologies) served as fiducial markers in all experiments to correct any lateral drift as described before (40). Coverslips in Attofluor cell chambers (Life Technologies) were imaged immediately after preparation in PBS (to detect paGFP using photoactivated localization microscopy, PALM) or direct stochastic optical reconstruction microscopy (dSTORM) imaging buffer (41) (to detect AF647, using dSTORM).

### 2.8. Optical setup, image acquisition, and data analysis

PALM and dSTORM imaging were performed using a 3D N-STORM super-resolution microscope system (32). Data was acquired using NIS Elements 4.3 software and ANDOR Solis 4.23. Images of 256 × 256 pixels (27 μm × 27 μm for PALM and 41 μm × 41 μm for dSTORM) were collected with a frame rate of 100 ms (PALM) and 10 ms (dSTORM). paGFP, which is a monomeric optical highlighter protein with good signal to noise ratio, was activated and excited with a 488 nm laser with power values ranging from 1.5 to 2 mW (measured out of the optical fiber) and a 505/15 emission filter. paGFP molecules were imaged until the signal was completely exhausted—typically 15,000–25,000 frames were acquired. For fluorescently labeled trastuzumab memAb (TmemAb-AF647), dSTORM imaging was performed similarly as before (32), with a laser power of 120 mW (measured out of the optical fiber) and acquiring 20,000–40,000 frames until the AF647 signal was exhausted. Additionally, NIS-Elements software was used with the following identification settings to capture positive signal and produce localization data for analysis: 700 as the minimum number of photons/localization, 200 nm minimum localization width, 400 nm maximum localization width, 300 nm initial fit width, 1.3 maximum axial ratio, and 1 pixel maximum displacement.

We characterized the blinking behavior of paGFP and TmemAb-AF647. As described previously (31, 32), the average number of localizations was approximately 5 for paGFP, whereas the average number of localizations for TmemAb-AF647 was approximately 3. These values represent the average number of localizations (discrete appearances α) of the fluorescent probe for a given set of imaging conditions and particular optical setup (31, 40, 42). We have demonstrated that robust molecular counting can be obtained using this approach (31, 32).

Pair correlation (PC) analysis and k-means-like clustering analysis were performed using MATLAB (The Mathworks, Inc., Natick, MA, USA) on 10–18 μm^2^ regions of interest (ROIs) as described previously (31, 32, 40, 42, 43). To briefly summarize key steps in the analysis, images of cells were first binarized using localization xy-coordinate centers obtained from NIS-elements. Localizations corresponding to noise (precision values outside the 98th percentile) were removed from these images. Individual ROIs were placed across the cells and the total number of localizations from within these regions was divided by a constant value (α, respective to the specific fluorophore) to obtain detected densities in terms of the number of molecules. Auto-correlation functions were computed using fast Fourier transforms to obtain the number of molecules per cluster and cluster radius (40, 42). Subsequently, localization precision and the cluster radius from PC analysis were directed into a k-means-like clustering algorithm (32, 43) to determine the fraction of clustered receptors. This calculation groups detected localizations via thresholds for spatial parameters (PC cluster radius and average localization precision) and a temporal parameter (maximum fluorophore dark time). Molecules were counted as part of a cluster (more than two receptors) if these spatiotemporal requirements are met; otherwise, molecules were counted as unclustered. All codes for this analysis workflow have been provided previously (32, 42) and the described approach was validated using Monte Carlo simulations (32).

The calculation for all *p*-values was performed in Excel using the Student’s *t*-test with a one-tailed distribution and heteroscedastic two-sample unequal variance type.

### 2.9. Drug treatment for Western blot assays

Cells were treated with PBS as vehicle control, 10 μM afatinib (LC LABS) for the indicated times. For multivalent meditope treatment, cells were treated with 10 nM trastuzumab memAb for 10 min, washed with warm media briefly and subsequently treated with either 10 nM Fc, 10 nM M2Fc, or 10 nM M4Fc for the indicated times. For parenteral antibody treatment, cells were treated with 10 nM trastuzumab and 10 nM pertuzumab in combination for the indicated times. The meditope treated and parental antibody treated cells were then washed briefly with warm media and treated with or without 10 ng/ml EGF (Genscript). All drug incubations were performed at 37°C in a cell culture incubator. To test for HER2-paGFP transfection activity, cells were treated with 10 ng/ml EGF for 30 min.

### 2.10. Kinetic studies

Cells were treated with multivalent meditopes as described above for the indicated times. Cells were lysed for immunoblotting with phospho-Akt, total Akt, phospho-HER2, total HER2, phospho-Erk1/2, total Erk1/2, phospho-EGFR, and total EGFR. Blots were imaged and quantified using the Image Lab software (Biorad). Akt, Erk1/2, and EGFR phosphorylation at each time point was quantified and normalized by calculating the ratio of pAkt over total Akt, pErk1/2 over total Erk1/2, and pEGFR over total EGFR, respectively, in each lane. The relative phosphorylation was normalized to the maximum response by control Fc at each time point. The experiment was repeated five times independently to calculate an average normalized relative phosphorylation.

### 2.11. Western blot

To prepare protein extracts for immunoblotting, cells were pelleted by centrifugation and washed two times using ice-cold PBS. The pellets were subsequently resuspended in lysis buffer (150 mM sodium chloride, 50 mM Tris, pH 8.0, 1% NP-40, Protease and Phosphatase Inhibitor Mini Tablets, Pierce) and rotated at 4°C for 30 min to lyse the cells. The cells were then centrifuged for 20 min at 10,000 rpm at 4°C. The supernatant consisting of the protein lysate was stored at −80°C. SDS-polyacrylamide gel electrophoresis and western blotting procedures were carried out using the treated cell lysates as per standard protocols. Primary antibodies used included anti-phospho-EGFR (Tyr 1068) (rabbit monoclonal, Cell Signaling), anti-EGFR (rabbit monoclonal, Abcam), anti- phospho-HER2 (tyr877) (rabbit monoclonal, Abcam) anti-HER2 (rabbit monoclonal, Abcam), anti-phospho-Akt (S473) (rabbit polyclonal, Abcam), anti-Akt (rabbit polyclonal, Abcam), anti-phospho-Erk1/2 (Thr202/Tyr204) (rabbit monoclonal, Cell Signaling); anti-Erk1/2 (rabbit polyclonal, Abcam); and anti-β-actin (mouse monoclonal, Cell signaling). Proteins were detected with Pierce ECL detection reagents (Pierce). The blots were imaged on a Biorad Chemidoc imager.

## 3. Results

### 3.1. Evaluation of a functional HER2-paGFP construct

In SMLM, target molecules of interest are detected via fluorescent reporters. Since one of our objectives was to use qSMLM to map the membrane organization of HER2 in breast cancer cells, we genetically tagged HER2 with photoactivatable green fluorescent protein (HER2-paGFP) and expressed this construct in MDA-MB-468 cells. This breast cancer cell line has a very low expression level of HER2 (32) and high expression of EGFR (44) and is considered HER2 negative. We then compared levels of HER2-paGFP expressed in MDA-MB-468 cells to native HER2 found in HER2 overexpressing SK-BR-3 breast cancer cells. According to our results from Western blot analysis (Supplementary Figure 1), the amount of expressed HER2-paGFP in MDA-MB-468 cells was comparable to endogenous HER2 in SK-BR-3 cells. In addition, both cell lines were exposed (for 0, 5, 30 min) to one of two treatments: either epidermal growth factor (EGF) or a combination of EGF + afatinib. EGF is a ligand for EGFR and has been shown to stimulate growth and differentiation, activate HER2, and promote phosphorylation of receptor tyrosine kinases. Conversely, afatinib is an irreversible inhibitor of both EGFR and HER2. During the treatments, we followed the phosphorylation status of five protein kinases involved in HER2 signaling: HER2, EGFR, protein kinase B (Akt), and extracellular signal-regulated kinases 1 and 2 (Erk1/2). Treating both cell lines with 10 ng/ml EGF led to the phosphorylation of all four kinases. Phosphorylation was reduced when both cell lines were additionally treated with 10 μM afatinib.

We next assessed how the phosphorylation of EGFR (Y1068) was affected by HER2-paGFP expression. Using Western blot analysis (Supplementary Figure 2), we probed the phosphorylation of EGFR when 10 ng/ml EGF was applied to the following cells: SK-BR-3, MDA-MB-468, MDA-MB-468 expressing HER2-paGFP, and MDA-MB-453 cells. MDA-MB-453 breast cancer cells do not express significant amounts of EGFR and HER2. As expected, MDA-MB-453 cells did not show EGFR phosphorylation following EGF treatment. Treatment of the other three cell lines with EGF showed increased phosphorylation of EGFR. This increase was more pronounced in MDA-MB-468 expressing HER2-paGFP than in MDA-MD-468 cells, which have minimal endogenous HER2. Altogether, results in Supplementary Figures 1, 2 suggest that the HER2-paGFP construct is functional.

### 3.2. Therapeutic antibodies affect clustering of HER2

**FIGURE 1:**
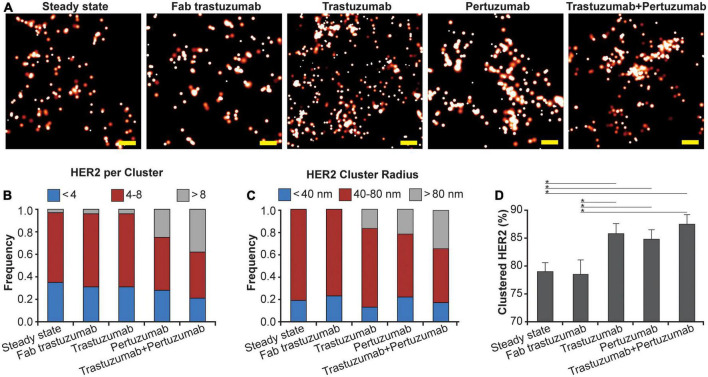
Effect of mAbs on HER2 nanoscale organization. **(A)** SMLM images of HER2-paGFP within a region from MDA-MB-468 cells in steady state and upon mAb/Fab treatment. An example for steady state HER2 is shown on the left, and examples for the organization of HER2 upon 10-min treatment with 20 nM Fab trastuzumab, 10 nM trastuzumab, 10 nM pertuzumab, and co-treatment with 10 nM trastuzumab and 10 nM pertuzumab are shown from left to right. All treatments were performed at 37°C. Scale bars: 200 nm. Standard PALM image analysis was employed (68) and localizations were grouped using a maximum blinking time of 5 s for paGFP and group radius of three times the maximum localization precision. **(B)** Distribution of detected HER2-paGFP molecules per cluster in steady state and upon mAb treatment. **(C)** Distribution of cluster radius of HER2-paGFP in steady state and upon mAb treatment. **(D)** Fraction of clustered HER2-paGFP molecules with SEM; *denotes *p* value ≤ 0.05. Quantitative analysis **(B–D)** was based on the following cell and region of interest (ROI) statistics: steady state (12 cells, 26 ROI), Fab trastuzumab (12 cells, 26 ROI), trastuzumab (13 cells, 30 ROI), pertuzumab (13 cells, 36 ROI), and co-treatment with trastuzumab and pertuzumab (12 cells, 29 ROI). ROIs for analysis were 10-18 μm^2^. While Fab trastuzumab had minimal effect on the HER2 distribution, mAbs and mAb combination induced significant clustering.

Using qSMLM, we assessed the distribution of HER2-paGFP in cultured MDA-MD-468 cells. As shown in Figure 1, HER2 was imaged both in the steady state and upon the following four treatments: Fab trastuzumab, trastuzumab, pertuzumab, and the combination of trastuzumab + pertuzumab. While some degree of HER2 clustering was observed in both the steady state and upon treatment with Fab trastuzumab, an increase in clustering was evident for all mAb treatments (Figure 1A). We assessed the imaging data using analysis algorithms to define the distribution of detected HER2 molecules per cluster (Figure 1B), the distribution of HER2 cluster radii (Figure 1C), and the fraction of clustered HER2 molecules (Figure 1D). The detected HER2 densities are shown in Supplementary Figure 3A and corresponding localization precision distributions are shown in Supplementary Figure 4A. Individual mAb treatments, trastuzumab or pertuzumab, resulted in an increased frequency of larger clusters with radii > 80 nm (Figure 1C, in gray). Trastuzumab + pertuzumab treatment produced the highest frequency of larger clusters (>80 nm radii) that contained > 8 HER2 molecules (Figures 1B, C, in gray). Additionally, the fraction of clustered HER2 molecules increased when breast cancer cells were treated with trastuzumab, pertuzumab, or a combination of trastuzumab + pertuzumab. Overall, the combination of trastuzumab + pertuzumab led to the most pronounced increase in HER2 clustering.

### 3.3. Clustering of HER2 increases upon treatment with multivalent reagents

Meditopes are cyclic 12-mer peptides that bind tightly (∼400 nM) to a unique site within the Fab arm of cetuximab (34). Previously, we demonstrated that this meditope-binding site is absent in human mAbs but can be readily grafted onto them; we termed the constructs meditope-enabled Abs (memAbs). We have demonstrated that the presence of the meditope does not affect antigen binding of memAbs (35–38). Here, we used the technology, in concert with qSMLM, to determine how the clustering of HER2 was affected by the valency. To this end, we fused the meditope sequence to either the N-termini or both the N- and C-termini of an IgG1 Fc (CH_2_-CH_3_) domain. We used this approach to generate two multivalent meditopes: Fc-divalent meditope (M2Fc) and Fc-tetravalent meditope (M4Fc).

**FIGURE 2:**
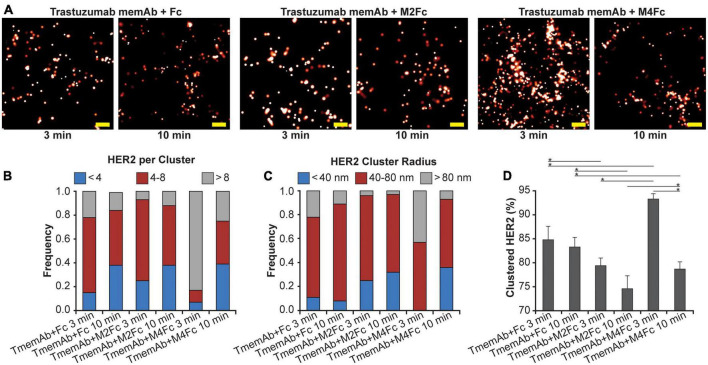
Effect of multivalent meditope-based ligands on HER2 nanoscale organization. **(A)** SMLM images of HER2-paGFP from a region on MDA-MB-468 cells upon treatment with trastuzumab memAb (TmemAb) in combination with Fc or Fc-multivalent meditiope constructs. Image pairs from left to right show HER2 upon treatment with 10 nM TmemAb for 10 min and subsequent incubation for either: 3 or 10 min with Fc; Fc-divalent meditope (M2Fc); or Fc-tetravalent meditope (M4Fc). All treatments were performed at 37°C and cells were briefly washed with warm media before treatment with Fc or Fc-meditope constructs. Scale bars: 200 nm. Standard PALM image analysis was employed (68) and localizations were grouped using a maximum blinking time of 5 s for paGFP and a group radius of three times the maximum localization precision. **(B)** Distribution of detected HER2-paGFP molecules per cluster upon treatment with TmemAb in combination with Fc or Fc-multivalent meditiope treatment. **(C)** Distribution of cluster radius of HER2-paGFP upon treatment with TmemAb in combination with Fc or Fc-multivalent meditiope treatment. **(D)** Fraction of clustered HER2-paGFP molecules with SEM; *denotes *p* value ≤ 0.05. Quantitative analysis **(B–D)** was based on the following cell and region of interest (ROI) statistics: TmemAb and Fc treatment for 3 min (12 cells, 27 ROI), TmemAb and Fc treatment for 10 min (12 cells, 26 ROI), TmemAb and M2Fc treatment for 3 min (12 cells, 28 ROI), TmemAb and M2Fc treatment for 10 min (14 cells, 34 ROI), TmemAb and M4Fc treatment for 3 min (12 cells, 30 regions), TmemAb and M4Fc treatment for 10 min (12 cells, 28 ROI). ROIs for analysis were 10–18 μm^2^. Treatment with M4Fc for 3 min had a significant clustering effect on the HER2 distribution.

Using qSMLM, we identified how the clustering of HER2-paGFP in MDA-MB-468 cells was affected by trastuzumab memAb (TmemAb) in combination with one of the following ligands: Fc domain (monovalent control), M2Fc (divalent meditope ligand), and M4Fc (tetravalent meditope ligand). Treatments were assessed at 3 and 10 min by qSMLM (Figure 2A and Supplementary Figure 5). The detected HER2 densities are shown in Supplementary Figure 3B and the associated localization precision distributions are shown in Supplementary Figure 4B. We assessed the imaging data upon each treatment using the same analysis as the therapeutic antibodies to obtain quantitative information on the distribution of detected HER2 per cluster (Figure 2B), the distribution of HER2 cluster radii (Figure 2C), and the fraction of clustered HER2 (Figure 2D). While some HER2 clustering was observed for all treatments at 3 min, it was most pronounced for tetravalent meditope M4Fc combined with TmemAb. This combination produced the highest frequency of larger-sized clusters (>80 nm radii) occupied with >8 HER2 molecules (Figures 2B, C in gray), and the highest fraction of clustered HER2 (Figure 2D). While still present at 10 min, the magnitude of HER2 clustering was reduced across all treatments.

### 3.4. Endogenous HER2 in BT-474 breast cancer cells shows a high degree of clustering upon treatment with multivalent reagents

In addition to mapping the nanoscale organization of HER2-paGFP, where HER2 is covalently attached to the fluorescent reporter, we assessed the organization of endogenous HER2 in breast cancer BT-474 cells. To this end, TmemAb was covalently labeled with a suitable fluorescent reporter, Alexa Fluor 647 (AF647), for qSMLM. For steady state, cells were first fixed and subsequently stained with TmemAb-AF647 postfixation (PF). To assess the effects of multivalent ligands, live cells were first incubated with reagents and subsequently fixed. In the latter scenario, we tested the following six treatments: (1) TmemAb-AF647 alone; (2–5) TmemAb-AF647 + one of four M2Fc ligands; and (6) TmemAb-AF647 + M4Fc. In all cases, TmemAb-AF647 was administered at a 10 nM concentration. In the case of M2Fc, the length of the meditope linker was varied (c10, c20, c30, and c37) to probe the impact of meditope geometry on HER2 clustering.

**FIGURE 3:**
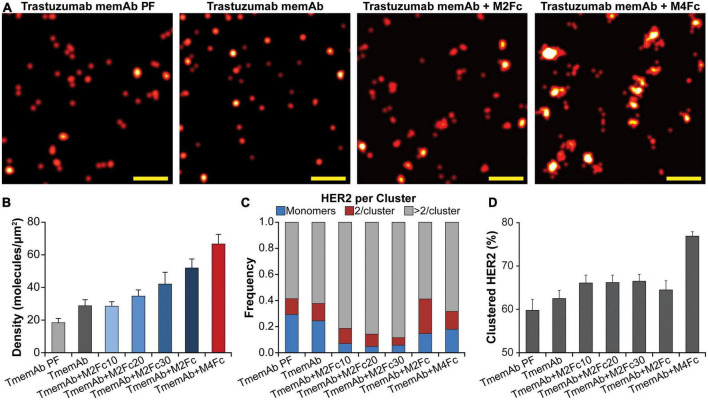
Trastuzumab memAb and multivalent meditopes induce HER2 reorganization. **(A)** SMLM images of AF647 labeled trastuzumab memAb (TmemAb) targeting HER2 on BT-474 cells. Live cells were incubated with 10 nM TmemAb-AF647, alone or premixed with 10 nM multivalent meditope (i.e., M2Fc and its variants or M4Fc), for 10 min at 37°C and subsequently fixed. Alternatively, cells were stained with 10 nM TmemAb-AF647 postfixation (PF). Scale bars: 200 nm. **(B)** Average molecular density of HER2 detected with TmemAb; the p values are provided in Supplementary Table 1. **(C)** Fraction of TmemAb-bound HER2 identified as an isolated receptor (blue), within a cluster of two receptors (red), or as part of a cluster with more than two receptors (gray). **(D)** Percentage of clustered proteins within a given ROI; the p values are provided in Supplementary Table 2. Quantitative analysis **(B–D)** used the following cell and region of interest (ROI; 18 μm^2^) statistics: TmemAb PF (14 cells, 29 ROI), TmemAb (14 cells, 34 ROI), TmemAb + M2Fc10 (16 cells, 40 ROI), TmemAb + M2Fc20 (17 cells, 39 ROI), TmemAb + M2Fc30 (15 cells, 35 ROI), TmemAb + M2Fc (23 cells, 43 ROI), and TmemAb + M4Fc (24 cells, 57 ROI).

SMLM images in Figure 3A illustrate that M2Fc and M4Fc induce an increase in HER2 clustering in combination with the TmemAb. Figure 3B summarizes the average detected molecular density of HER2 (as detected with TmemAb-AF647) and the corresponding localization precision distributions are provided in Supplementary Figure 4C. The p values for the HER2 densities are given in Supplementary Table 1. The highest HER2 densities were detected when TmemAb-AF647 was paired with either M2Fc (c37) or M4Fc (Figure 3B). According to studies in different cell lines, the normal function of HER2 may be influenced by local differences in HER2 spatial arrangement on the cell membrane: monomers, dimers, and clusters (29–33). We thus analyzed the imaging data to determine nanoscale organization of endogenous HER2 upon multivalent ligand treatment, Figures 3C, D. We identified the fraction of TmemAb-bound HER2 as either an isolated receptor (monomer), a cluster of two receptors, or a cluster with more than two receptors; the p values for the fraction of clustered HER2 are given in Supplementary Table 2. Based on the analysis (Figure 3C), HER2 monomers were most abundant when TmemAb-AF647 was used alone (PF or in live cells) and lower frequencies of HER2 monomers were observed when TmemAb-AF647 was combined with multivalent meditopes. By far the highest percentage of clustered HER2 was observed for the tetravalent meditope (Figure 3D).

### 3.5. Combination of memAbs and multivalent meditopes significantly reduces receptor tyrosine kinase phosphorylation at early time points

**FIGURE 4:**
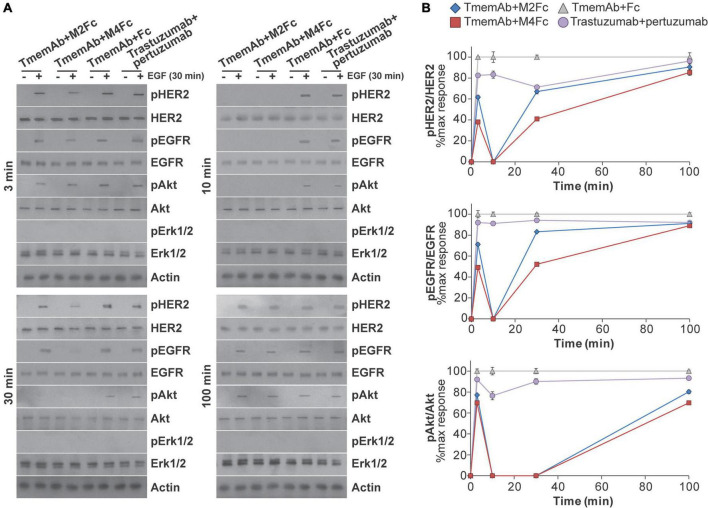
Effects of multivalent meditopes on cell signaling pathways upon EGF stimulation of HER2-paGFP transfected MDA-MB-468 cells. Cells were treated with 10 nM trastuzumab memAb for 10 min, followed by either 10 nM Fc, M2Fc, M4Fc or Fc for indicated times. Cells were incubated for additional 30 min without or with addition of 10 ng/ml EGF. Alternatively, cells were co-treated with 10 nM trastuzumab and 10 nM pertuzumab for indicated periods of time. After brief wash with warm media, cells were incubated for additional 30 min without or with addition of 10 ng/ml EGF. All treatments were performed at 37°C; cells were briefly washed with warm media before and after treatment with Fc or Fc-meditope constructs. **(A)** Phosphorylation of EGFR(Y1068), HER2(Y877), and downstream signaling targets Akt(S473) and Erk1/2(p42/44 T202/Y204) was determined using Western blot analysis for cells treated as described above. **(B)** Relative phosphorylation of Akt, HER2, and EGFR for cells treated as described above was calculated as the ratio of pAkt over total Akt, pHER2 over total HER2 or pEGFR over total EGFR and normalized to the maximum response by Fc at the indicated time points. Results are representative of 5 independent experiments. Colors show Fc treatment in gray, M2Fc treatment in blue, M4Fc in red, and combined parental trastuzumab and pertuzumab in purple. M4Fc has the highest inhibition of EGF mediated phosphorylation.

Given these findings, we were interested in identifying how multivalent treatments affected downstream HER2 signaling pathways. For example, data suggests HER receptors may be highly expressed in a trastuzumab resistant setting (45–52) and may associate into signaling platforms to activate pathways and compensate for trastuzumab-induced inhibition (45, 52). We thus tested how exposing MDA-MB-468 cells that express HER2-paGFP to different treatments affected the phosphorylation of HER2, EGFR, Akt, and Erk1/2. Western blot analysis was used to identify the impact of four different treatments: (1) TmemAb + Fc; (2) TmemAb + M2Fc; (3) TmemAb + M4Fc; and (4) trastuzumab + pertuzumab. After the treatments were administered for the indicated times (Figure 4A), cells were incubated for an additional 30 min at 37°C either in the presence or absence of EGF. Images of the Western blots are shown in Figure 4A. The relative phosphorylation of Akt, HER2, and EGFR was calculated as a ratio (pReceptor divided by total Receptor) and normalized to the maximum response by Fc (Figure 4B). Remarkably, both TmemAb + M2Fc and TmemAb + M4Fc effectively blocked EGFR, HER2, and Akt phosphorylation at 10 min. Compared to the control (TmemAb + Fc), phosphorylation of EGFR, HER2, and Akt was attenuated with TmemAb and multivalent meditope ligands at 30 min; the effect was more pronounced with M4Fc. Compared to the control, phosphorylation of EGFR, HER2, and Akt was attenuated with trastuzumab + pertuzumab at 10 and 30 min, but to a lower extent.

### 3.6. Treatment with TmemAb + tetravalent meditope activates antibody dependent cellular cytotoxicity

**FIGURE 5:**
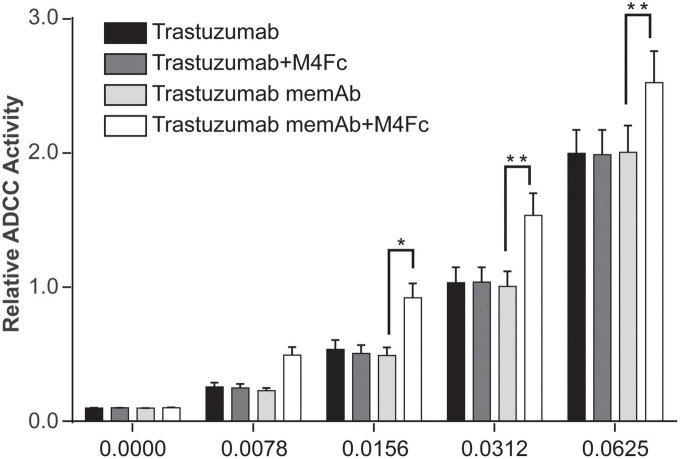
Effects of M4Fc on ADCC in SK-BR-3 cells. ADCC activity was measured in SK-BR-3 cells treated with increasing doses of trastuzumab or trastuzumab memAb in the presence or absence of 10 nM M4Fc for 6 h. Data was compiled from three experiments and analyzed using a 2-way ANOVA (*p < 0.05; **p < 0.01). Data is normalized to the untreated (0 nM antibody) cell signal. Treatment with M4Fc in the presence of trastuzumab memAb shows higher levels of ADCC activity.

One mechanism promoted by trastuzumab, which may lead to improved clinical outcomes, is ADCC (15, 16). Similarly, pertuzumab has been shown to activate ADCC and the enhanced efficacy of the antibody combination treatment has been explained, in part, by this mode of action (9, 17). Since clustering and phosphorylation were significantly increased by the tetravalent meditope, we were interested in measuring how it affected ADCC. To this end, SK-BR-3 cells were exposed for 6 h at 37°C to one of four treatments: (1) trastuzumab; (2) trastuzumab + M4Fc; (3) TmemAb; (4) TmemAb + M4Fc. The concentration of M4Fc was held at 10 nM while we assessed different concentrations of the Abs. Consistently across the various concentrations (Figure 5), the highest levels of ADCC activity were observed when cells were treated with TmemAb + M4Fc. A control experiment (Supplementary Figure 6) was performed, which demonstrated that ADCC activity was negligible without target or effector cells. Overall, these results suggest that the multivalent meditope, in combination with meditope-enabled trastuzumab, enhances ADCC activity.

## 4. Discussion

While the precise mechanism of mAb combinations is still being investigated, trastuzumab plus pertuzumab combined with chemotherapy is now part of the clinician’s armamentarium to treat HER2-positive breast cancer. Multiple mechanisms have been presented to explain its clinical utility, including its ability to block (homo- and hetero-) HER2 dimerization, increase ADCC, and/or attenuate the HER2 signaling cascade (9, 17, 26–28). Recent studies have demonstrated additional effects of HER2 associated with receptor nanoscale organization. For example, high expression levels of HER2 on breast cancer cells appear to drive individual receptors into detectable molecular clusters and potentially altering interactions with adjacent cells (30). Large HER2 clusters also appear to be more resistant to internalization when activated (53–57). Of interest, abnormal trafficking into intracellular compartments appears to be a common theme for cell surface receptors involved in tumor development (58, 59). Indeed, HER2 has been detected within nanometer sized cholesterol-enriched plasma membrane domains (60–62) that support rapid signaling. Additionally, membrane HER2 nanoscale clustering is sensitive to treatment with targeted or chemotherapeutic agents (31) and may be associated with therapy response in HER2-positive breast cancer (33). These studies suggest there is a potential link between important physiological events, such as the membrane residency time of HER2, and local differences in the membrane organization of HER2. Given that the propagation of signals *in vitro* occurs on minute time scales (Supplementary Figures 1, 2), we probed the molecular dynamics of HER2 membrane organization at early time points. We show that mAb(s)/memAb-ligand treatments affect cell surface receptor clustering; the observed effects were dependent on ligand valency. Moreover, these treatments also affected downstream processes, including phosphorylation in HER2 signaling pathways, at notably short timeframes.

To understand the effects of mAb and memAb therapies on HER2 molecular organization, we combined meditope technology, which allows us to control the valency of the treatment, with qSMLM. These technologies enabled us to report on the effect of valency on molecular density and organization of HER2. Because SMLM imaging requires a fluorescent reporter, we applied both PALM (detecting HER2 genetically tagged with optical highlighter protein, in this case, paGFP) and dSTORM (detecting HER2 tagged with an affinity reagent that contains a fluorescent dye, in this case, AF647) to comprehensively assess HER2 clustering. HER2-paGFP was transiently overexpressed in MDA-MB-468 breast cancer cells (very low levels of endogenous HER2). Having established the functionality of the construct (Supplementary Figure 1, 2), we set out to map how the membrane organization of HER2-paGFP was affected by either clinical therapies or our meditope reagents. For the therapeutic antibodies, the images show that the most extensive clustering of HER2 (Figure 1) was produced when cells were treated with a combination of pertuzumab + trastuzumab. For the meditope reagents, the most significant clustering of HER2 (Figure 2) was observed when cells were treated with TmemAb plus the M4Fc. Similar results were obtained when we used fluorescently labeled trastuzumab to detect native HER2 in BT-474 breast cancer cells (Figure 3A): by far the highest fraction of clustered HER2 was observed for the tetravalent meditope (Figure 3D). Of note, trastuzumab can likely bind both sterically accessible dimeric HER2 and monomeric HER2 (63). However, trastuzumab cannot bind—and thus detect—HER2 that is sterically hindered (e.g., with heavily glycosylated proteins) or HER2 that lacks an extracellular trastuzumab binding domain. Accordingly, using fluorescently labeled trastuzumab we have previously reported (33) differences in HER2 clustering in cell lines that have a different expression of large, glycosylated proteins (e.g., JIMT-1 cells vs. SK-BR-3 cells). Thus, trastuzumab can sensitively detect changes in HER2 clustering at different receptor densities and local membrane environments.

In the canonical mode of signaling, cell surface dimerization of HER receptors leads to interaction between their intracellular kinase domains, transphosphorylation of tyrosine residues in the C-terminal ends, and initiation of signals that are transduced to the nucleus via different pathways including mitogen-activated protein kinases (MAPKs), phosphoinositide-3-kinase (PI3K)/Akt, and phospholipase C gamma (PLCgamma) pathways. Interestingly, activated HER2 can resist significant endocytosis (54). While trastuzumab-induced receptor downregulation is a slow process, it can affect the remodeling of the plasma membrane at early time points (preceding endocytosis) (64). Given that mAb treatment (with or without multivalent ligands) can quickly alter HER2 nanoscale organization, we next probed if these biologics can also affect the propagation of signals. We show that changes to HER2 organization at early time points are accompanied by changes in physiological function. The multivalent meditopes (complexed with TmemAb) that show the most pronounced HER2 clustering also exhibited the highest inhibition of EGF-mediated phosphorylation (Figure 4). Additionally, tetravalent meditope/TmemAb enhanced ADCC (Figure 5).

In addition to canonical signaling, HER fragments can signal directly. Such fragments are typically generated by the action of alpha and gamma secretases. For example, previous work has shown gene transcription to be regulated by a nuclear carboxy-terminal fragment comprising the cytoplasmic domain of HER4 (65, 66) and HER2 (67). Importantly, a fragment of the intracellular domain of HER2, termed 611-CTF (carboxy terminal fragment), can constitutively homodimerize and regulate MET, EPHA2, matrix metalloproteinase 1, interleukin 11, angiopoietin-like 4, and different integrins, promoting mammary tumor growth and metastasis. Future experiments are needed to explore if the membrane nanoscale organization of HER receptors is associated with fragment generation and activity.

Altogether, our results suggest that high valent treatments, achieved either through a combination of clinical Abs or meditope technology, have the capacity to arrange HER2 on the membrane of breast cancer cells, may abrogate elements of the HER2 signaling cascade, and may ultimately lead to the elimination of breast cancer cells. This study as well as others highlight the role of valency on receptor dynamics and geography. The multivalent ligand approach may be a general strategy for manipulating receptor clustering applicable to many unique targets (e.g., PD-L1, CD38, CD19). Beyond increasing the valency, this approach also vitiates the need to identify additional mAbs to non-overlapping epitopes. However, the consequences of massive clustering will be unique for each system and need to be assessed. Critically, this study also suggests that qSMLM can provide valuable information and guide the effort to design multivalent biologics (e.g., bispecific and biparatropic mAbs) for therapeutic intent.

## Data availability statement

The raw data supporting the conclusions of this article will be made available by the authors, without undue reservation.

## Author contributions

DLW, OG, RJ, SB, JCW, and TJ-T conceived and designed the experiments and developed experimental/computational techniques. DLW, OG, RJ, SB, KM, KNA, CZ, SJT, EEC, JCW, and TJ-T carried out the experiments and analyzed the data. DLW, OG, RJ, SB, KM, KNA, SJT, IJT, JCW, and TJ-T wrote the manuscript. All authors contributed to the article and approved the submitted version.
