# Supplementary material for: Using quantitative single molecule localization microscopy to optimize multivalent HER2-targeting ligands
Source: Front Med (Lausanne). 2023 Apr 17;10:1064242. doi: 10.3389/fmed.2023.1064242 (PMC10149953; doi:10.3389/fmed.2023.1064242)
Supplement: Supplementary file 1 [file Data_Sheet_1.pdf]

Supplementary Material

1 Supplementary Methods

Meditope Fc sequences

Divalent Meditope-Fc (M2FC)
linker: 37 aa

```
GCQFDLSTRRLRCGGSRSGGTSGGGSVPGSGSSGSTSGSGKSSEGSGQASTHTCPPCPAPELLGGPSVFLFPPKPKDTLMI
SRTPEVTCVVVDVSHEDPEVKFNWYVDGVEVHNAKTKPREEQYNSTYRVVSVLTVLHQDWLNGKEYKCKVSNKALPAPIEK
TISKAKGQPREPQVYTLPPSRDELTKNQVSLTCLVKGFYPSDIAVEWESNGQPENNYKTTPPVLDSDGSFFLYSKLTVDKS
RWQQGNVFSCSVMHEALHNHYTQKSLSLSPGK
```

Tetravalent Meditope-Fc (M4FC)
first linker: 39 aa
second linker: 30 aa

```
GCQFDLSTRRLRCGGSRSGGTSGTGSTSGSGSSGSTSGSGKSSEGSGQASKGTHTCPPCPAPELLGGPSVFLFPPKPKDTL
MISRTPEVTCVVVDVSHEDPEVKFNWYVDGVEVHNAKTKPREEQYNSTYRVVSVLTVLHQDWLNGKEYKCKVSNKALPAPI
EKTISKAKGQPREPQVYTLPPSRDELTKNQVSLTCLVKGFYPSDIAVEWESNGQPENNYKTTPPVLDSDGSFFLYSKLTVD
KSRWQQGNVFSCSVMHEALHNHYTQKSLSLSPGKGGSRSGGTSSSSSGSGSGSSGSTSGSGSSGCQFDLSTRRLRCG
```

2 Supplementary Figures

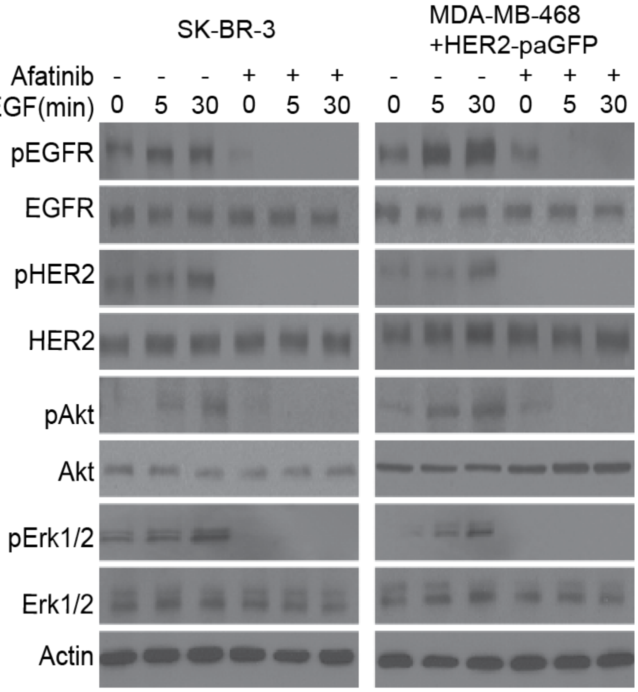

1.1 Supplementary Figure 1. HER2-paGFP construct is functional.

SK-BR-3 and HER2-paGFP transfected MDA-MB-468 cells were treated with EGF in a time-dependent manner in the presence and the absence of the irreversible EGFR/HER2 inhibitor afatinib. SK-BR-3 and MDA-MB-468 cell lines express EGFR (IHC 2+ and 3+, respectively) (1); SK-BR-3 cells express HER2 (IHC 3+) but MDA-MB-468 cells do not express high amounts of HER2 (IHC 0) (1). Phosphorylation of HER2(Y877), EGFR(Y1068), Akt(S473), and Erk1/2(p42/44 T202/Y204) was monitored. In both cell lines, 10 ng/mL EGF induced phosphorylation of tyrosine kinases, while 10 µM afatinib reduced or abolished phosphorylation of tyrosine kinases. Loading quantities were confirmed using antibodies specific for HER2, EGFR, Akt, Erk1/2, and Actin.

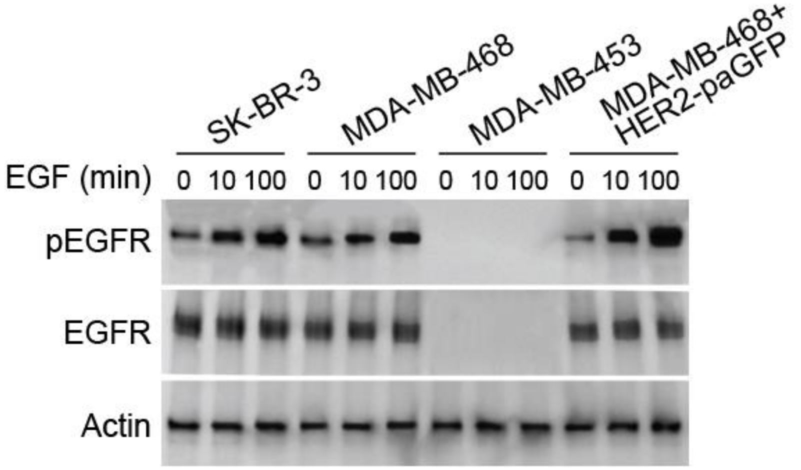

1.2 Supplementary Figure 2. EGFR phosphorylation in breast cancer cell lines.

SK-BR-3, MDA-MB-468, MDA-MB-453, and MDA-MB-468 cells expressing HER2-paGFP were treated with 10 ng/mL EGF for the indicated amount of time and the phosphorylation of EGFR was examined by Western blots using antibodies specific for phospho-EGFR(Y1068). HER2-paGFP transfection increases EGFR phosphorylation at later time points as expected from a functional HER2 construct (2).

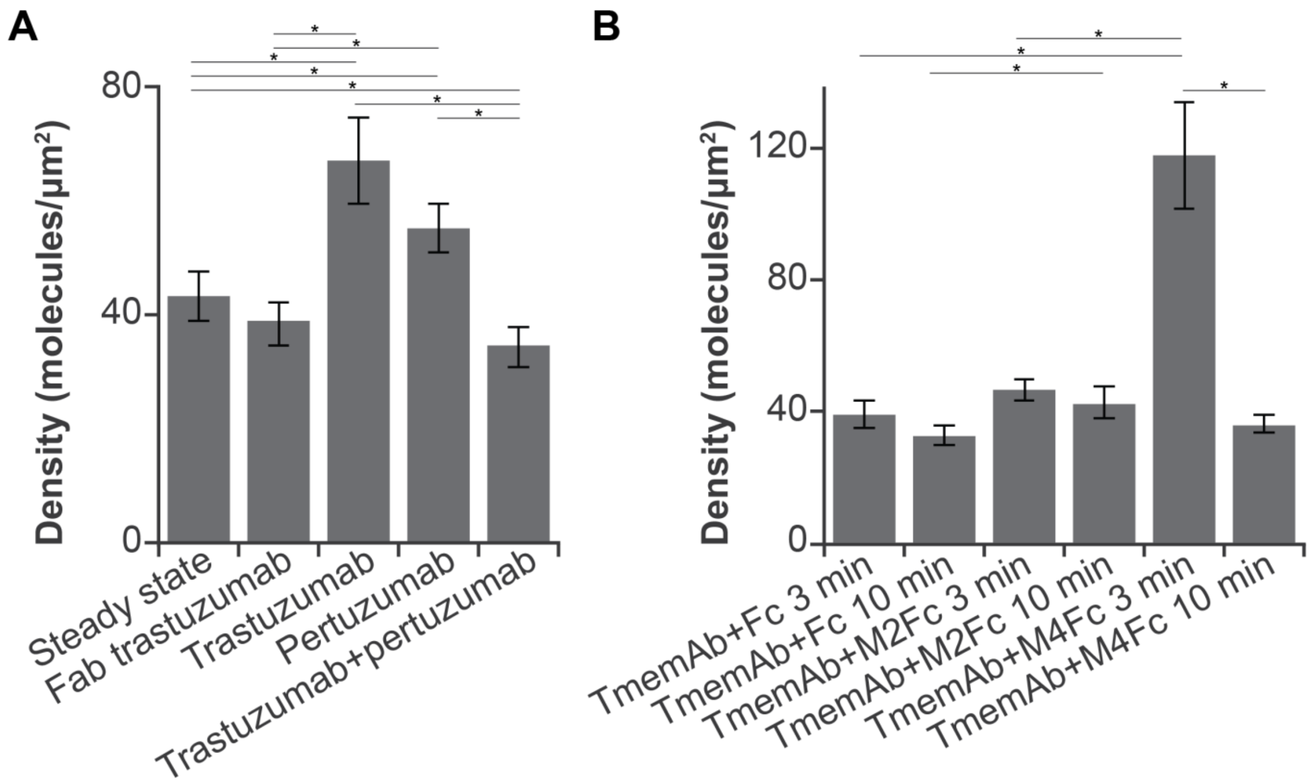

1.3 Supplementary Figure 3. Detected density values of HER2-paGFP.

(**A**) Density of HER2-paGFP in steady state and upon mAb/Fab treatment. (**B**) Density of HER2-paGFP upon treatment with trastuzumab memAb (TmemAb) in combination with Fc or Fc-multivalent meditiope constructs. Average values for all regions with SEM are shown. * denotes p value ≤ 0.05.

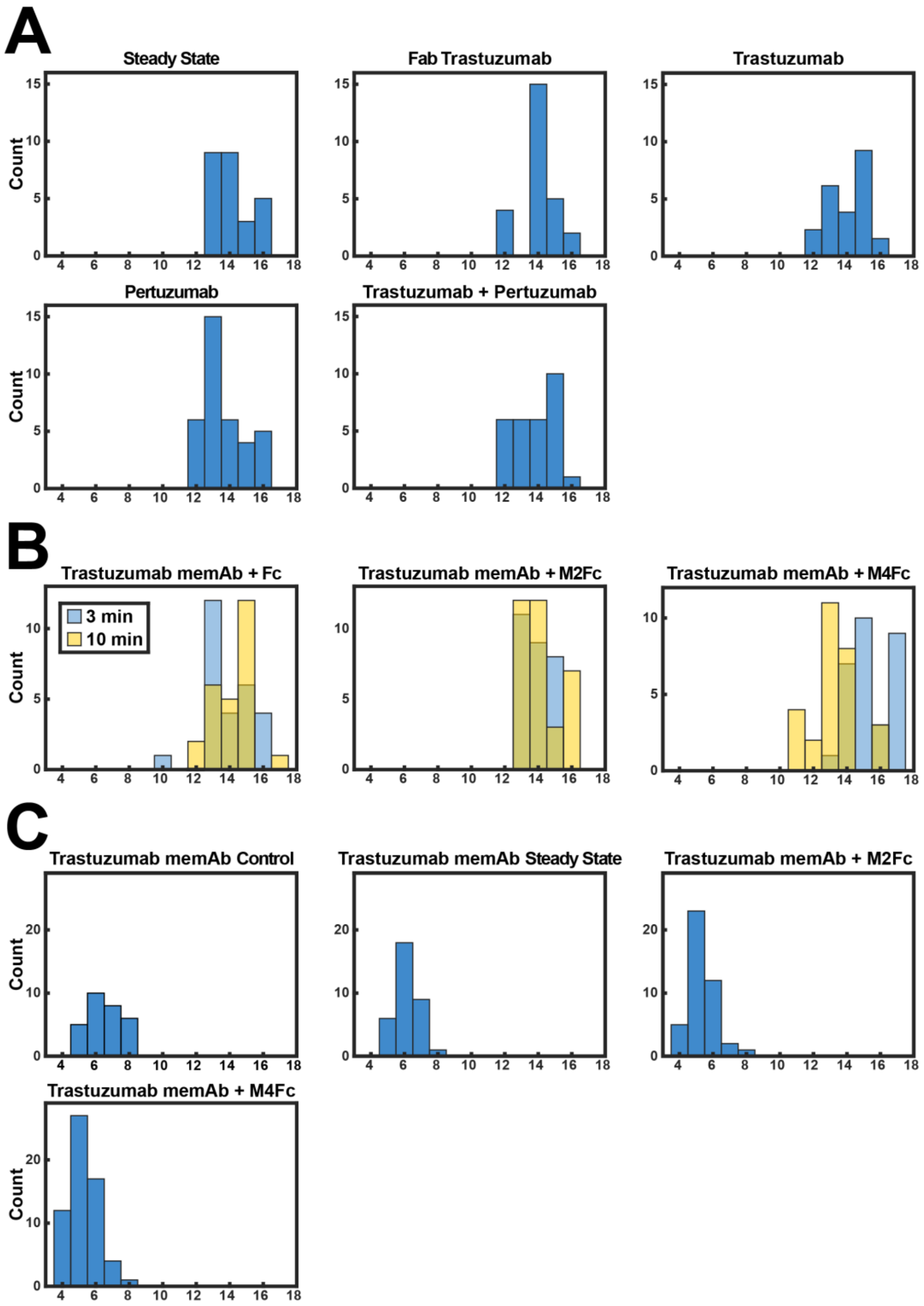

1.4 Supplementary Figure 4. Localization precision distributions.

Histograms display all ROI localization precision (nm) data presented in (**A**) Fig. 1, (**B**) Fig. 2, and (**C**) Fig. 3.

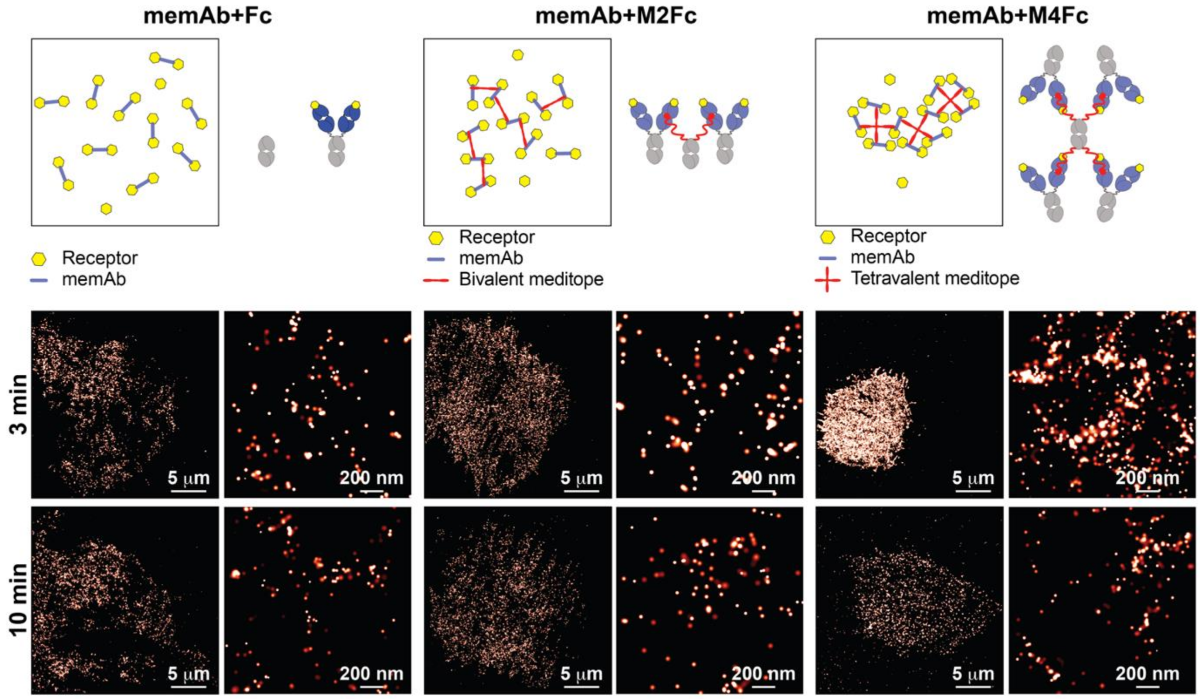

1.5 Supplementary Figure 5. HER2 clustering with multivalent ligands. Top, scheme; **Bottom,** single molecule localization microscopy images.

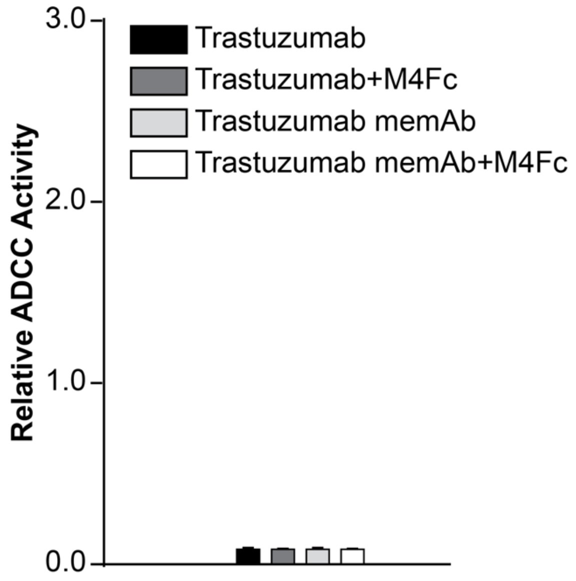

1.6 Supplementary Figure 5. ADCC control experiment.

ADCC activity was measured without target or effector cells with 1 nM mAbs with or without 10 nM M4Fc. Data is normalized to the untreated (0 nM mAb) signal with target and effector cells included.

3 Supplementary Tables

Supplementary Table 1. All p values for average molecular density of HER2 in Figure 3B.

	TmemAb	10aa	20aa	30aa	37aa	M4Fc
**TmemAb PF**	1.9E-06	7.0E-07	6.8E-07	3.4E-08	6.8E-18	4.4E-19
**TmemAb**		0.458	0.053	0.003	3.4E-09	4.3E-14
**10aa**			0.037	0.002	7.5E-10	2.4E-14
**20aa**				0.056	7.4E-06	1.0E-11
**30aa**					0.023	1.7E-07
**37aa**						1.6E-05

Supplementary Table 2. All p values for fraction of clustered HER2 in Figure 3D.

	TmemAb	10aa	20aa	30aa	37aa	M4Fc
**TmemAb PF**	0.0026	5.8E-05	2.8E-05	9.2E-06	0.055	1.8E-15
**TmemAb**		0.015	0.059	0.042	0.025	6.8E-11
**10aa**			0.31	0.28	0.024	1.3E-10
**20aa**				0.49	0.011	2.9E-08
**30aa**					0.0076	1.7E-09
**37aa**						4.6E-11

Reference

1. Subik K, Lee J-F, Baxter L, Strzepek T, Costello D, Crowley P, et al. The Expression Patterns of Er, Pr, Her2, Ck5/6, Egfr, Ki-67 and Ar by Immunohistochemical Analysis in Breast Cancer Cell Lines. *Breast Cancer: Basic and Clinical Research* (2010) 4:35-41.

2. Offterdinger M, Bastiaens PI. Prolonged Egfr Signaling by Erbb2-Mediated Sequestration at the Plasma Membrane. *Traffic* (2008) 9(1):147-55. Epub 2007/10/25. doi: 10.1111/j.1600-0854.2007.00665.x.
